# The Outcome of Allogeneic Hematopoietic Stem Cell Transplantation From Different Donors in Recipients With Mucopolysaccharidosis

**DOI:** 10.3389/fped.2022.877735

**Published:** 2022-06-30

**Authors:** Yuhua Qu, Hao Liu, Likun Wei, Shushan Nie, Wenjiao Ding, Sha Liu, Haiyan Liu, Hua Jiang

**Affiliations:** ^1^Department of Hematology and Oncology, Guangzhou Women and Children’s Medical Center, Guangzhou Medical University, Guangzhou, China; ^2^Department of Orthopedics, The Central Hospital of Wuhan, Tongji Medical College, Huazhong University of Science and Technology, Wuhan, China; ^3^Department of Stomatology, The Central Hospital of Wuhan, Tongji Medical College, Huazhong University of Science and Technology, Wuhan, China

**Keywords:** allogeneic hematopoietic stem cell transplantation, umbilical cord blood transplantation, conditioning regimen, mucopolysaccharidoses, outcomes

## Abstract

There is limited information regarding hematopoietic stem cell transplantation (HSCT) for mucopolysaccharidosis (MPS) IV and VI. This study examined the full donor chimerism, specific lysosomal enzyme levels, and the survival of different MPS children after HSCT from various donor sources and compared the prognosis. A total of 42 children with MPS underwent HSCT, 9 cases were type I, 14 were type II, 15 were type IV, and 4 were type VI. A total of 24 patients received peripheral blood stem cells (PBSC) and 18 patients received umbilical cord blood (UCB). Patients who received PBSC were conditioned with intravenous (IV) busulfan every 6 h for a total of 16 doses, IV cyclophosphamide (CY, 200 mg/kg), and antihuman thymocyte globulin (ATG, 10 mg/kg). While conditioning regimen of patients who received UCB was adjusted to ATG (preposed, pre-) + busulfan + fludarabine + Cy, which includes IV ATG (pre-, 6 mg/kg), IV busulfan every 6 h for a total of 16 doses, IV fludarabine (200 mg/m^2^) and CY (200 mg/kg). Also, 95.2% (40 of 42) of patients achieved full donor chimerism, and all patients’ specific lysosomal enzyme levels reached normal. The estimated overall survival (OS) at 1 year was 92.9%. There was no significant difference in 1-year OS between patients who received PBSC transplantation and those who received UCB grafts (87.5% vs. 100%, *p* = 0.0247). The incidence of acute and chronic GVHD did not differ between them. The incidences of pneumonia in PBSC recipients and UCB recipients were 45.8 and 33.3%, respectively, but there few patients suffering from respiratory failure (4.2 and 5.6%, respectively) due to pneumonia. The incidence of cytomegaloviremia was also high in both groups, 58.3 and 44.4% respectively, However, no patient developed CMV disease. All deaths (3 of 42) occurred in patients receiving PBSC grafts, and there was no death in patients receiving UCB grafts. There was no death after transplantation in patients with MPS IV and VI. In addition, respiratory and nervous system functions were improved, whereas valvular heart disease was improved in some patients but progressed in more patients after transplantation. In summary, HSCT is a good therapeutic option for MPS, not only for patients with MPS I or II but also for those with MPS IV or VI. The specific lysosomal enzyme levels can be completely restored to normal, which is the basis for patients to resolve a broad range of clinical outcomes. Moreover, UCB with suitable HLA (HLA-match above 7/10 and 4/6) is a suitable donor source for MPS. Patients who underwent UCB transplantation using the conditioning regimen ATG (pre-) + busulfan + fludarabine + Cy can achieve a higher proportion of full donor chimerism and survival with less severe complications. HSCT can improve organs function in patients with MPS, but it is still worth exploring.

## Introduction

Mucopolysaccharidoses (MPS) are a group of lysosomal storage disorders (LSDs) caused by genetic defects. These congenital defects lead to a lack or deficiency of enzymes, which results in progressive lysosomal accumulation of glycosaminoglycans (GAGs) followed by the development of various somatic and neurologic symptoms, including coarse facial features, cognitive retardation, hepatosplenomegaly, cardiac abnormalities, dysostosis multiplex, hernias, corneal clouding, and loss of developmental milestones (1). All of them are autosomal-recessive-inherited diseases except type II (an X-linked inherited disease) (2). Although the severity of clinical manifestations varies, most patients present disability and often die at a young age.

Due to the relentlessly progressive nature of the disease, it has long been recognized that early diagnosis and treatment of MPS in the asymptomatic stage may effectively preserve organic function and improve outcomes. However, delayed diagnosis is common on account of insidious onset and limitations of sensitive laboratory index. The current treatment target of MPS is to slow the progression of MPS and improve quality of life. In practice, two primary treatments for patients with MPS are available; hematopoietic stem cell transplantation (HSCT) and enzyme replacement therapy (ERT) (3). In the former, endogenous enzymes are produced by engrafted donor leukocytes, while in the latter, exogenous enzymes are taken up to host tissues that remain genetically enzyme-deficient. ERT has been approved to treat MPS I, II, IVA (elosulfase alpha), VI, and VII (vestronidase alpha), which can effectively improve the clinical symptoms of patients with MPS while reducing treatment-related complications. However, ERT cannot cross the blood–brain barrier (BBB), and thus, cannot improve neurological symptoms; moreover, ERT can cause an immune response against the infused enzyme in the recipient’s body, which can result in compromised treatment outcomes. Finally, ERT is a lifelong treatment, which increases its cost. While with HSCT, healthy donor cells are transplanted, donor stem cells circulate into the bloodstream, crossing the BBB and differentiating (macrophage, microglia, etc.). The microglial cells can secrete the deficient enzyme to the different parts of the brain to improve neurological symptoms, making HSCT advantageous over ERT (4).

Since the first successful HSCT in 1980, HSCT has been considered the standard of care in children with MPS IH and an optional treatment for Hurler/Scheie syndrome (MPS IH/S) and Scheie syndrome (MPS-IS) (attenuated phenotypes of MPS I), MPS II, MPS IVA, MPS VI, and MPS VII (2–8). Rodgers et al. (6) reported that HSCT has increased survival in MPS IH beyond the third decade of life, especially in improving transplant strategies since 2004, resulting in better long-term survival in the current patient population. In a recent study, the 5-year overall survival and event-free survival have been reported to be 95.2 and 90.3%, respectively (9). In addition, recipients of HSCT can acquire donor cells from three different sources, namely, bone marrow, peripheral blood (PB), or umbilical cord blood (UCB) (3). In recent years, UCB has become a popular donor source because of the better donor chimerism achieved. However, few studies documenting outcomes between different types of MPS and the influence of varying donor sources on them. Hence, this study examined the full donor chimerism, specific lysosomal enzymes levels, the overall survival, and organ function of different MPS children after HSCT from different donor sources and compared the prognosis.

## Patients and Methods

### Patients

From December 2013 to July 2020, a total of 42 children with MPS underwent HSCT at the Guangzhou Women and Children’s Medical Center and had follow-up for ≥1 year after HSCT. Among them, 9 cases were type I, 14 were type II, 15 were type IV, and 4 were type VI. A total of 28 were boys and 14 were girls. The median age at transplantation was 36 months (range, 11–108 months). There were 6 cases ≤24 months old, 20 cases were between 24 and 48 months old, and 16 were over 48 months old. The median time from diagnosis to transplantation was 7 months (range, 0.5–48 months). All patients who met the diagnostic criteria included a significant decrease in the level of one of the following lysosomal enzymes: serum α-L-iduronidase, iduronate sulfatase, galactose-6-sulfatase, β-galactosidase, or arylsulfatase B; positive urine GAGs; and had been excluded multiple sulfatase deficiency and GM1 ganglioside disease. A total of 37 patients had undergone genetic testing. Information on demographic characteristics, pretransplantation history, physical examination, routine laboratory tests, imaging data primary diagnosis, preparative regimens, stem cell source, type of donor (sibling vs. unrelated), agents used for graft-versus-host disease (GVHD) prophylaxis, and complications and management were collected. Written informed consent was obtained from the parents or legal guardians of the patients.

This study was performed in accordance with the modified Helsinki Declaration, and the protocol was approved by the Ethics Committee of Nanfang Hospital before study initiation. All the recipients and/or guardians provided written informed consent prior to study enrollment.

### Transplantation (Donor Selection, Conditioning Regimen, Supportive Care, and Monitoring)

For the HSCT donor/graft source, 24 patients received peripheral blood stem cells (PBSC). Among them, 4 from matched family donor, 10 from matched unrelated donor, 4 from mismatched unrelated donor (7/10 to 9/10 HLA-matched), and 6 from haploid donor (2 donors were MPS carrier); 18 patients received UCB, including 1 from matched family fresh cord blood, 15 from mismatched unrelated cord blood (7/10 to 9/10 HLA-matched), and 2 from double mismatched unrelated cord blood (1 from 6/10 to 8/10 HLA-matched, the other from 6/10 to 7/10 HLA-matched). Patients who received PBSC were conditioned with intravenous (IV) busulfan every 6 h for a total of 16 doses, IV cyclophosphamide (CY, 200 mg/kg),and antihuman thymocyte globulin (ATG, 10 mg/kg). In contrast, the conditioning regimen of patients who received UCB was adjusted. The conditioning regimen for the first patient who underwent UCB transplantation was the same for patients with PB HSCT. The second patient who underwent UCB transplantation was conditioned with IV ATG [preposed (pre-), 6 mg/kg], IV CY (120 mg/kg), and busulfan every 6 h for a total of 16 doses. The conditioning regimen for all the following patients who underwent UCB transplantation was ATG (pre-) + busulfan + fludarabine (FLU) + Cy, which includes IV ATG (pre-, 6 mg/kg), IV busulfan every 6 h for a total of 16 doses, IV FLU (200 mg/m^2^), and CY (200 mg/kg). GVHD prophylaxis regimen consisted of cyclosporine (CSA), mycophenolate mofetil (MMF), and methotrexate (MTX). CSA (2.5 mg⋅kg^–1^ day^–1^) was given as an infusion starting on day −1 and then intake once orally became feasible with a target blood level of 150–250 ng⋅ml^–1^. CSA was continued as the full dose for 100 days and then gradually withdrawn. The dosage of MMF was 0.25 g for every 12 h and administered orally from day −1 to day 30 after transplantation. IV MTX 15 mg⋅m^2^ was given on day +1 and then 10 mg⋅m^2^ on days +3 and +6. Antimicrobial prophylaxis consisted of acyclovir, cotrimoxazole, and posaconazole. Cytomegalovirus (CMV) and Epstein–Barr virus (EBV) DNA were monitored weekly until 100 days post-engraftment. After transplantation, donor hematopoietic chimerism monitoring was performed on whole blood by analysis of short tandem repeat polymerase chain reaction (STR-PCR). The specific lysosomal enzyme level and urine GAGs were routinely assessed at 1, 3, 6, 9, and 12 months, and thereafter, once every 6 months after transplantation. In addition, organ functions were surveyed every half year. Intellectual disability was confirmed by the Gesell Developmental Scale (for children aged under 6 years) or the revised Wechsler Intelligence Scale for Children (for children aged over 6 years), whose developmental quotient (DQ)/intelligence quotient was equivalent to or less than 70.

### Statistical Analysis

Descriptive statistics of subject characteristics were summarized separately for the entire cohort by transplant donor subgroups (PBSC and UCB). Median and ranges were reported for continuous covariates with frequency and percentage for categorical variables. Comparisons of descriptive characteristics were based on the *t*-test or chi-square tests for continuous and categorical variables, respectively. Probabilities of overall survival (OS) were calculated using the Kaplan–Meier estimate, and the two-sided log-rank test was used for univariate comparisons. All *p*-values quoted are two-sided, with a level of significance of 0.05. Statistical analyses were performed using the SPSS 23 software.

## Results

### Patients’ Characteristics and Clinical Manifestations

A total of 42 children with MPS were included. Patients and transplantation characteristics are shown in Table 1. Also, 47.6% of the patients were between 24 and 48 months of age at final HSCT, with 14.3% less than 24 months of age and 38.1% more than 4 years of age. Matched unrelated donor and UCB were the predominant donor and stem cell source, respectively. Due to the lack of suitable donors, two of the haploid donors were carriers of MPS. Various conditioning regimens were used with the large proportion of patients receiving a myeloablative conditioning therapy comprising IV busulfan, IV CY (200 mg/kg), and ATG (10 mg/kg). Using the above regimen, the first patient who received UCB suffered from repeated pneumonia and cytomegaloviremia due to too intense immunosuppression of the conditioning regimen and slow recovery of hematopoiesis. So, we prepositioned ATG (ATG pre-, the usage time was changed from −4, −3, and −2 days to −9, −8, and −7 days), reduced the accumulated dose of ATG to 6 mg/kg and Cy dose to 120 mg/kg, and unchanged BU for the second patient who received UCB. However, this patient did not achieve complete donor chimerism. Therefore, we deemed that the conditioning regimen was not immunosuppressive enough. On this basis, FLU (200 mg/m^2^) was introduced, the dose of Cy was increased to 200 mg/kg, and ATG (pre-, 6 mg/kg) and busulfan (every 6 h for a total of 16 doses) remained unchanged with the subsequent conditioning regimen for the follow-up patients who received UCB. GVHD prophylaxis regimen for most of the patients (*n* = 23) who received PBSCs was CSA, MMF, and MTX; however, for the majority of patients (*n* = 16), who received UCB, only CSA was used to prevent GVHD. The follow-up period after HSCT was a median of 3.1 years (range 1.0–7.6 years).

**TABLE 1:** Patient and transplantation characteristics (*N* = 42).

**Patient characteristics**	
Male, n(%)	28(66.7)
Median age at transplant, months	36(11,108)
Median time from diagnosis to transplantation, months	7(0.5,48)
**Age at transplant, n (%)**	
<24 months	6(14.3)
24 −<48 months	20(47.6)
≥48 months	16(38.1)
**MPStype**	
MPS I	9(21.4)
MPS II	14(33.3)
MPS IV	15(35.7)
MPS VI	4(9.5)
**Donor characteristics and stem cell source, n (%)**	
PBSC	24(57.1)
Matched family donor	4(9.5)
Matched unrelated donor	10(23.8)
Mismatched unrelated donor	4(9.5)
Haploid donor	6(14.3)
UCB	18(42.9)
Matched family umbilical cord blood	1(2.4)
Matched unrelated umbilical cord blood	0(0)
Mismatched unrelated umbilical cord blood(HLA 7/10-9/10)	15(35.7)
Double mismatched unrelated umbilical cord blood	2(4.8)
Conditioning regimen, n (%)	
Bu + Cy + ATG	25(59.5)
ATG(Pre-) + Bu + Cy	1(2.4)
ATG(Pre-) + BU + FIU + Cy	16(38.1)
GVHD prophylaxis, n (%)	
CSA alone	16(38.1)
CSA + MTX	3(7.1)
CSA + MMF + MTX	23(54.8)
**Time of neutrophil implantation (days after transplantation, +d)**	
PBSC	+13 d(+12 to +16 d)
UCB	+15 d(+11 to +31 d)

*MPS, mucopolysaccharidosis; PBSC, peripheral blood stem cells; UCB, umbilical cord blood; GVHD, graft-versus-host disease; ATG, anti-thymocyte globulin; Pre-, preposed; Bu, busulfan; FIU, fludarabine; CSA, ciclosporin; Cy, cyclophosphamide; MMF, mycophenolate mofetil; MTX, methotrexate.*

### Chimerism and Specific Lysosomal Enzyme Levels

**FIGURE 1:**
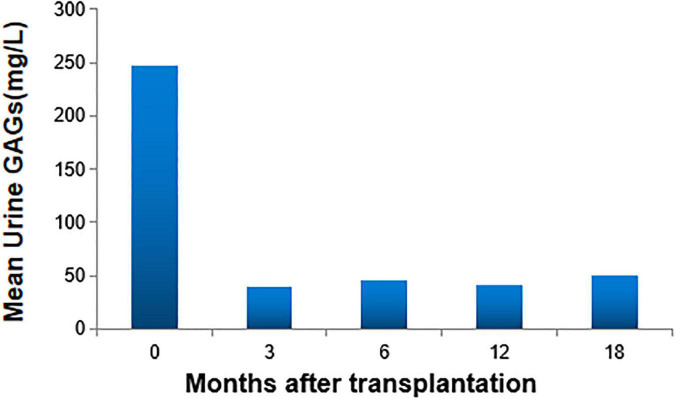
Urine glycosaminoglycans (GAGs) levels. At 3 months after transplantation, the patient’s urine GAGs level was significantly lower than that before transplantation, and it remained at a stable low level since then.

A total of 40 patients (95.2%, 40 of 42) achieved full donor chimerism (Table 2). All (100%, 24 of 24) patients who received PBSC grafts achieved full donor chimerism. While 88.9% (16 of 18) of patients with UCB grafts attained complete donor chimerism, 2 achieved stable mixed chimerism (donor chimerism rate fluctuated from 87 to 94%). After transplantation, the specific lysosomal enzyme levels reached normal in all patients (100%) in both UCB recipients and PBSC recipients, even in two patients whose donors were haploid carrier donors. No significant differences in full donor chimerism or normal enzyme levels were observed between patients who received UCB and PBSC grafts (*p* = 0.178 and *p* = 1.000, respectively). The urine GAGs levels in all patients were significantly reduced. At 3 months after transplantation, the patient’s urine GAGs level was significantly lower than that before transplantation, and it remained at a stable low level since then as shown in Figure 1.

**TABLE 2:** Chimerism, enzyme activity, and transplantation-related complications (*N* = 42).

Outcome	PBSC, *n* = 24	UCB, *n* = 18	*P*-value
Full donor chimerism n (%)	24(100)	16(88.9)	0.178
Normal enzyme level	24(100)	16(100)	1.000
Mixed chimerism n (%)	0	2(11.1)	0.178
Normal enzyme level	0	2(100)	1.000
aGVHDn (%)	11(45.8)	10(55.5)	0.533
Grade I-II	10(41.7)	8(44.4)	0.857
Grade III-IV	1(4.2)	2(11.1)	0.567
cGVHDn (%)	2(8.3)	1(5.6)	1.000
Mild	1(4.2)	0	1.000
Moderate	1(4.2)	1(5.6)	1.000
Severe	0	0	1.000
**Other transplantation-related complications n (%)**			
Cytomegaloviremia	14(58.3)	8(44.4)	0.372
EB-viremia	5(20.8)	0	0.06
Pneumonia	11(45.8)	7(38.9)	0.653
Respiratory failure	1(4.2)	1(5.6)	1.000
VOD	0	1(5.6)	0.429
TMA	3(12.5)	2(11.1)	1.000
PRES	0	1(5.6)	0.429
Hemolytic anemia	2(8.4)	0(0)	0.429
Hemorrhagic cystitis	1(4.2)	0	1.000
**Causes of death n (%)**	3(12.5)	0	0.247
Grade III and IV GVHD	1(4.2)	0	1.000
TMA	1(4.2)	0	1.000
Grade III and IV GVHD + TMA	1(4.2)	0	1.000

*cGVHD, chronic GVHD; CMV, cytomegalovirus; EB, Epstein–Barr virus; VOD, hepatic venous-occlusive disease; TMA, thrombotic microangiopathy; PRES, posterior reversible encephalopathy syndrome.*

### Survival Probability and Transplantation-Related Complications After Hematopoietic Stem Cell Transplantation With Peripheral Blood Stem Cells Compared to Umbilical Cord Blood

**FIGURE 2:**
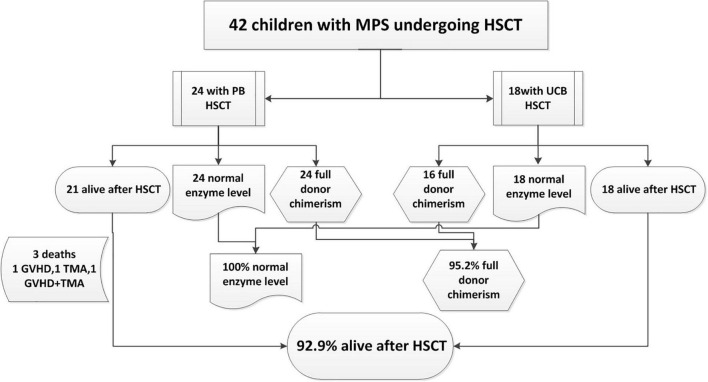
Outcome of 42 children with mucopolysaccharidosis (MPS) undergoing hematopoietic stem cell transplantation (HSCT) from December 2013 to July 2020.

**FIGURE 3:**
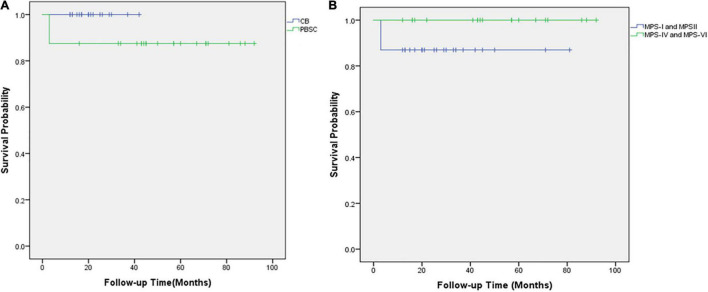
The overall survival of 42 children with mucopolysaccharidosis after hematopoietic stem cell transplantation. **(A)** Kaplan–Meier survival curves after HSCT for those who received UCB grafts (blue line) and those who received PBSC transplantation (green line). **(B)** Kaplan–Meier survival curves after HSCT for MPS types I and II (blue line) and MPS types IV and VI (green line).

The outcome of the 42 children after HSCT is illustrated in Figure 2. The 1-year survival rate was 92.9%. The estimated OS at 1 year was 92.9%. The 1-year survival curve of patients receiving PBSC and UCB grafts is shown in Figure 3. The 1-year survival curves of patients receiving UCB were significantly better than patients receiving PBSC (*p* = 0.002). Transplantation-related complications are shown in Table 2. The incidence of grade I to II acute GVHD (aGVHD) was 41.1% (10 of 24) and 44.4% (8 of 18), respectively, in PBSC recipients and UCB recipients, and the incidence of grade III and IV aGVHD was 4.2% (1 of 24) and 11.1% (2 of 18), and the incidence of mild-to-moderate chronic GVHD was 8.3% (2 of 24) and 5.6% (1 of 18), respectively. Whether patients who received PBSC or those who received UCB, the incidence of pneumonia (45.8 and 33.3%, respectively) was high, which may be related to the airway characteristics of patients with mucopolysaccharide and previous infections. The incidence of cytomegaloviremia in PBSC recipients and UCB recipients was 58.3 and 44.4%, respectively; however, no patient developed CMV disease. The incidence of Epstein–Barr viremia was relatively high in patients with PBSC transplantation [20.8% (5 of 24)], while none of the patients with UCB transplantation developed Epstein–Barr viremia; there was no significant difference between the two groups (*p* = 0.06). The incidence of TMA in the two groups was similar (12.5 and 11.1%, respectively, in PBSC recipients and UCB recipients), and there was no significant difference (*p* = 1.000). The incidence of other transplant-related complications such as hepatic venous-occlusive disease, posterior reversible encephalopathy syndrome, hemolytic anemia, and hemorrhagic cystitis is very low as shown in Table 2. The overall prognosis was good, with a high rate of OS and low incidence of transplant-related severe complications.

### Causes of Death

Of the three deaths after HCST, one child died of grade III and IV GVHD, one died of thrombotic microangiopathy, and one died of grade III and IV GVHD with thrombotic microangiopathy. The median time of death was 3 months (range, 3–4 months) after transplantation. There was no late death in our cohort. As shown in Figure 2, all deaths occurred in patients receiving PBSC grafts, and there was no death in patients receiving UCB grafts. There was no significant difference in mortality between patients with PBSC transplantation and those with UCB transplantation (*p* = 0.247). On log-rank analysis, neither pretransplant factors (age at transplantation and sex) nor transplant factors (conditioning regimen and donor) correlated with increased mortality in the patients (Table 3). Of the three deaths, one patient was type I and two were type II, while there was no death after transplantation in patients with MPS IV and VI. There was no significant difference in mortality between MPS I and II and MPS IV and VI (*p* = 0.239).

**TABLE 3:** Cox proportional hazards model results for survival over the first year post-HSCT (*n* = 42).

Variable	n (%)	95% CI	*p*-value
**Sex**			
Male	28(66.7)	0.685–3.662	0.283
Female	14(33.3)		
**Age at transplant**			
<24 months	6(14.3)	0.373–1.148	0.654
24 −<48 months	20(47.6)		
≥48 months	16(38.1)		
**MPStype**			
MPS I	9(21.4)	0.760–1.471	0.742
MPS II	14(33.3)		
MPS IV	15(35.7)		
MPS VI	4(9.5)		
**Donor characteristics and Stem cell source**			
PBSC	24(57.1)	0.001–8.655	0.292
UCB	18(42.9)		
**Conditioning regimen**			
Bu + Cy + ATG	25(59.5)	0.231–36.707	0.409
ATG(Pre-) + Bu + Cy	1(2.4)		
ATG(Pre-) + BU + FIU + Cy	16(38.1)		

*On log-rank analysis, neither pretransplant factors (age at transplantation and sex) nor transplant factors (conditioning regimen and donor) correlated with increased mortality in the patients.*

### Evaluation of Organ Function

At 6 years of follow-up, organs function outcome was assessed in 33 patients (type I: *n* = 6; type II: *n* = 12; type IV: *n* = 12; type VI: *n* = 3). Before transplantation, the most common clinical manifestations were upper-airway obstruction, frequent airway infection, heart valve disorders, and central nervous symptoms. After transplantation, upper-airway obstruction and frequent airway infection were significantly improved. Valvular heart disease was improved in some patients but progressed in more patients. Motor skills were significantly improved (83.3%) after transplantation, while speech skills showed less improvement (33.3%). The incidence of hydrocephalus was 12.1% before transplantation (including three cases of MPS II and one case of MPS I); two cases (MPS II) developed cervical cord compression and after that underwent decompression surgery; the other two (one case of MPS I and one case of MPS II) got improved (Table 4).

**TABLE 4:** Organs function and clinical outcomes in patients with MPS after transplantation (*n* = 33).

Organs function and clinical outcomes	Before HSCT	After HSCT		
		
	Present, n (%)	Effective, n (%)	No change, n (%)	Progressed n (%)
**Respiratory system**				
Upper-airway obstruction	23(69.7)	18(78.3)	5(21.7)	0
Frequent airway infection	12(36.4)	9(75.0)	2(16.7)	1(8.3)
Heart valve disorders	24(72.7)	4(12.1)	9(37.5)	11(45.8)
**Central nervous system**				
Motor	18(54.5)	15(83.3)	2(11.1)	1(5.6)
Speech	18(54.5)	6(33.3)	8(44.4)	4(22.2)
Hydrocephalus	4(12.1)	2(50.0)	0	2(50.0)

## Discussion

Since 1980, Hobbs et al. performed the first HSCT in a patient with MPS IH (10) and achieved good clinical improvement. More studies have proved that HSCT could cure MPS I. HSCT can enable endogenous enzyme donation by taking engrafted donor leukocytes up to host tissues that remain genetically enzyme-deficient. In the case of HSCT, donor-derived hematopoietic cells circulate into the bloodstream, which can cross the BBB and differentiate into microglia. The microglial cells secrete the deficient enzyme to the different parts of the brain (4). HSCT has been shown to improve CNS impairment in MPS I, II, and VII (2, 5, 8, 11, 12) and has become the standard of care therapy for patients with HS. However, HSCT treatment remains controversial in MPS IV and VI. In this study, we reported 42 patients with different types of MPS who underwent PBSC transplantation or UCB transplantation. The estimated 1-year overall survival rate was 92.9%, consistent with the results reported in patients with MPS I by Rodgers et al. The survival rate was improved compared with that of patients before 2014 (6). The increase in survival was related to improved management level in the peritransplantation period and the improved conditioning regimens, and the type of donor in recent years. This was consistent with the results of patients with MPS IH and MPS VI reported by the European Group for Blood and Marrow Transplantation and the Center for International Blood and Marrow Transplant Research (CIBMTR) (13, 14). In our study, patients with transplantation of UCB had higher survival rates (100%) than those with transplantation of PB (87.5%). UCB has become a popular donor source for patients of MPS in recent years. In 2005, the EBMT issued guidelines that the best donor sources are non-carrier HLA-matched family donors, the following best are matched unrelated cord blood (15). At this time, the main emphasis was placed on the status of HLA-matched family donors. After more than 10 years, Boelens et al. reported the best donor sources are identical antigen matched UCB or identically matched HLA siblings in 2016 (16). Rodger et al. discovered that the 8-year survival rate for recipients receiving related bone marrow transplantation (BMT) was similar to the recipient receiving unrelated UCB transplantation, which was higher than that for recipients receiving unrelated BMT in 2017 (6). These two reports had put HLA-matched UCB on an equal footing with HLA-matched PB or bone marrow. In our study, patients who received UCB showed better survival. Although the graft failure rate had decreased in recent years, aplastic-type graft failure was more common in UCB during the period, which was perceived to represent likely insufficient immune suppression for the recipient. Therefore, in addition to a good donor source, the conditioning regimen is also essential.

Unlike EBMT and CIBMTR, in this study, the rate of full donor chimerism was high (95.2%, 40 of 42). Only two patients who received UCB grafts achieved stable mixed chimerism. Our high ratio of full donor chimerism was likely connected with a busulfan-based myeloablative conditioning regimen was used. Boelens et al. reported that reduced-intensity conditioning regimens and T cell-depleted might be the critical factors for transplant failure of MPS transplantation (17). In contrast, a busulfan-based myeloablative conditioning regimen would protect against graft failure. They proposed that the best combination of regimen and donor source was a busulfan-based myeloablative conditioning regimen (18), an HLA-matched UCB donor, a non-carrier HLA-matched sibling, or a fully HLA-matched unrelated donor. Of our 42 patients, 18 patients underwent UCB transplantation, including 7 patients with type I, 7 patients with type II, 3 patients with type IV, and 1 with type VI. There were no deaths in all the patients who underwent UCB transplantation. The specific lysosomal enzyme levels returned to normal, even though two patients were not fully donor chimerism (stable mixed chimerism, donor chimerism fluctuated between 89 and 95%). No mortality of UCB transplantation depends on the preparative regimen. Our conditioning regimen of patients who received UCB went through the process of groping. The first patient who received UCB was conditioned with IV busulfan every 6 h for a total of 16 doses, IV CY (200 mg/kg) and ATG (10 mg/kg). However, she had five bouts of pneumonia after the transplant, and we speculated that the dose of ATG and Cy in the conditioning regimen may be too large, resulting in excessive immunosuppression after transplantation. Therefore, we preloaded and reduced ATG to 6 mg/kg in the second patient and reduced Cy to 120 mg/kg without changing busulfan. In this patient, there was no infection, but a new problem arose, the donor chimerism dropped to 89%. We believe that the conditioning regimen for the second recipient is inadequate. A combination of busulfan and FLU as a conditioning regimen was suggested as the most recent recommendation for successful engraftment in 2012. FLU is currently regarded as the preferred preparative regimen on account of lesser toxicity than CY when combined with busulfan and has been displayed to attain the same engraftment rate success in recipients (16, 18). Based on the literature reports (16, 18, 19) and our experience with the first two patients who underwent UCB transplantation, the conditioning regimen for all the following patients who underwent UCB transplantation was ATG (pre-) + busulfan + FLU + Cy, which includes IV ATG (pre-, 6 mg/kg), IV busulfan every 6 h for a total of 16 doses, IV FLU (200 mg/m^2^) and CY (200 mg/kg). Under this conditioning regimen, patients with MPS obtained good results (OS 100%) with full donor chimerism and a lower level of infection. The cumulative incidence of GVHD in this study is relatively high, but severe GVHD is rare; the PBSC and UCB groups were 4.2 and 11.1%, respectively. The incidence of GVHD may be related to the addition of FLU and preposed of ATG in conditioning regimen, but more clinical data are needed to confirm.

Although HSCT is widely used in patients with MPS types I and II (2, 5, 6) and has achieved good results, it remains controversial in patients with MPS types IV and VI. In 2014, Chinen et al. reported the first successful case report of HSCT in MPS IVA (20). Bone mineral density in the lumbar vertebrae (L2–L4) was increased by 50% at 1 year post-BMT, and pulmonary function was well-stabilized during 9 years post-BMT. In 2016, Yabe et al. reported HSCT cases in four patients with MPS IVA (20, 21). Transplantation was successful in all four cases with complete engraftment and without any severe GVHD. In 2019, Akyol et al. (22, 23) considered that HSCT could not be recommended for patients with MPS IVA due to the lack of evidence and may be an option for patients with MPS VI. The latter have a matched related donor (or unrelated donor) or cord blood graft. They also considered that due to the risk of mortality HSCT must be only performed in an institution with a multidisciplinary team experienced in the care of patients with MPS VI. In 2020, Kazuki et al. summarized that HSCT could improve pulmonary function, BMD, and the activities of daily living (ADL) and could reduce the frequency of surgical intervention in patients with MPS IVA, suggesting that HSCT could be a useful supportive treatment option for patients with MPS IVA (24). In our study, there were 15 patients with MPS IVA and 4 patients with MPS VI. Also, 94.7% (18 of 19) of the patients have achieved complete donor chimerism. All patients with MPS IVA and VI with HSCT have achieved normal enzyme activity levels without any severe complications, and all were alive from 2013 to the present. This study provides strong evidence for the application of HSCT in patients with MPS IVA and VI. In addition, this study assessed respiratory, cardiac, and nervous system functions before and after transplantation. Respiratory and nervous system functions were improved, whereas valvular heart disease was improved in some patients but progressed in more patients. These organ function assessments were consistent with other reports (2, 8). Even so, skeletal deformity and cardiac function are still worth investigating; the function of each organ of MPS after transplantation remains to be studied.

## Conclusion

The data from our 42 patients manifest that HSCT is a good therapeutic option for MPS, not only for patients with MPS I or II but also for those with MPS IV or VI. Patients’ specific lysosomal enzyme level could be completely restored to normal, which is the basis for resolving a broad range of clinical outcomes. Moreover, UCB with suitable HLA matching (7/10 to 10/10 HLA-matched; meanwhile, 4/6 to 6/6 HLA-matched) is a suitable donor source for MPS. Patients who underwent UCB transplantation using the conditioning regimen ATG + busulfan + FLU + Cy [includes IV ATG (preposed, 6 mg/kg), IV busulfan every 6 h for a total of 16 doses, IV FLU (200 mg/m^2^), and CY (200 mg/kg)] can achieve a higher proportion of full donor chimerism and survival with less severe complications. HSCT can improve organ function in patients with MPS, but it is still worth exploring.

## Data Availability Statement

The original contributions presented in this study are included in the article/supplementary material, further inquiries can be directed to the corresponding author.

## Ethics Statement

The studies involving human participants were reviewed and approved by the Medical Ethics Committee of Guangzhou Women and Children Medical Center. Written informed consent to participate in this study was provided by the participants’ legal guardian/next of kin.

## Author Contributions

YQ and HJ: conceptualization and funding acquisition. YQ, HL, LW, and WD: data curation. YQ, HL, LW, and SL: formal analysis. YQ, HL, and LW: investigation. YQ and HYL: methodology. YQ and WD: resources. HL and LW: software. HJ: supervision and writing – review and editing. YQ: writing – original draft. All authors read and approved the final manuscript.

## Conflict of Interest

The authors declare that the research was conducted in the absence of any commercial or financial relationships that could be construed as a potential conflict of interest.

## Publisher’s Note

All claims expressed in this article are solely those of the authors and do not necessarily represent those of their affiliated organizations, or those of the publisher, the editors and the reviewers. Any product that may be evaluated in this article, or claim that may be made by its manufacturer, is not guaranteed or endorsed by the publisher.
